# Standard Operating Procedure to Optimize Resazurin-Based Viability Assays

**DOI:** 10.3390/bios14040156

**Published:** 2024-03-26

**Authors:** Jessica Petiti, Laura Revel, Carla Divieto

**Affiliations:** Division of Advanced Materials Metrology and Life Sciences, Istituto Nazionale di Ricerca Metrologica (INRIM), 10135 Turin, Italy; l.revel@inrim.it (L.R.); c.divieto@inrim.it (C.D.)

**Keywords:** cell viability, resazurin assay, consistency of results, preclinical drug tests, protocol standardization

## Abstract

The resazurin assay, also known as the Alamar Blue assay, stands as a cornerstone technique in cell biology, microbiology, and drug development. It assesses the viability of cells through the conversion of resazurin into highly fluorescent resorufin. The resulting fluorescence intensity provides a reliable estimate of viable cell numbers. Cytotoxicity assays, such as the resazurin-based method, play a crucial role in the screening of potential drug candidates and in the assessment of pharmaceutical and chemical toxicity. In recent years, inconsistencies have arisen in pharmacogenomic studies, often due to poorly optimized laboratory protocols. These inconsistencies hinder progress in understanding how substances affect cell health, leading to unreliable findings. Thus, the need for standardized and rigorously optimized protocols is evident to ensure consistent and accurate results in cytotoxicity studies. This manuscript describes a standardized procedure for optimizing resazurin-based viability assays to improve the reliability of cytotoxicity data. This optimization approach focuses on critical experimental parameters and data quality, aiming to achieve a level of measurement imprecision of less than 20%. In conclusion, to address the critical issues of reproducibility and reliability, protocol standardization, such as the one described in this manuscript, can greatly enhance the credibility of cytotoxicity studies, ultimately advancing drug safety assessments.

## 1. Introduction

**Figure 1:**
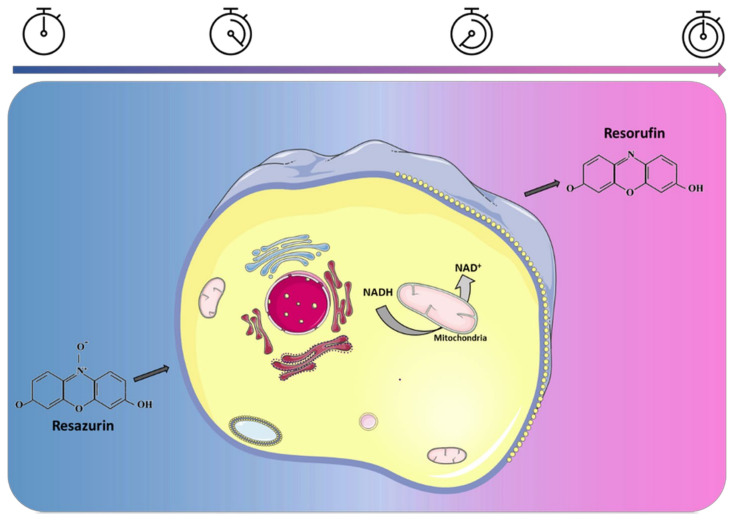
Resazurin reduction to resorufin in viable cells.

Resazurin, a nontoxic blue dye also known as Alamar Blue, is a widely used biochemical assay employed in various fields, such as cell biology, microbiology, and drug development [1]. This assay provides a rapid and sensitive method to assess the viability and metabolic activity of living cells. When cells are treated with resazurin, it is internalized by cells and metabolically reduced in the cytoplasm of living cells by cytosolic, microsomal, and mitochondrial dehydrogenase or reductase enzymes, producing resorufin, a highly fluorescent pink compound [2]. Resorufin is released from cells into the culture medium, which can be collected to measure its fluorescence intensity (FI) (Figure 1).

A direct correlation is established between the cell count and the FI. The interpolation of the FI data of an unknown sample using a calibration curve permits the estimation of the number of viable (living) cells.

The resazurin reduction assay is relatively inexpensive and more sensitive than tetrazolium assays [3]. While both assays rely on the reduction of a dye to a colored formazan product by metabolically active cells, there are key differences between the two. Tetrazolium salts, such as MTT (3-(4,5-dimethylthiazol-2-yl)-2,5-diphenyltetrazolium bromide) and MTS (3-(4,5-dimethylthiazol-2-yl)-5-(3-carboxymethoxyphenyl)-2-(4-sulfophenyl)-2H-tetrazolium), are reduced by cellular dehydrogenases to produce formazan crystals [4]. In contrast, resazurin is a cell-permeable blue dye that is reduced primarily by mitochondrial enzymes [2]. Mitochondria are highly active organelles within cells and play a central role in cellular metabolism [5]. As a result, even slight changes in cellular metabolic activity, which may not be detected using tetrazolium-based assays, can lead to measurable reductions in resazurin to resorufin conversion. Furthermore, resazurin exhibits a wider dynamic range of detection compared to tetrazolium-based dyes [6]. This means that the fluorescent signal generated by the reduction of resazurin is more proportional to changes in cell viability across a broader range of cell densities and metabolic states. Both these factors contribute to the enhanced sensitivity of the resazurin assay compared to tetrazolium-based assays. Additionally, resazurin is less affected by compounds that interfere with tetrazolium reduction, providing more reliable results in certain experimental conditions [7]. Another advantage of resazurin is that it is non-toxic to cells when used at low concentrations and for short incubation periods, making it suitable for time-lapse experiments.

Resazurin assays can be used for several purposes, such as drug discovery (drug safety and toxicity studies) and proliferation assays (growth factor or cytokine studies). In addition, this assay can be used to evaluate cell viability in different 3D matrices [8,9,10]. Drug development is one of the most important applications where the resazurin-based viability assay can play a pivotal role in ensuring the safety and efficacy of substances that interact with living cells. Indeed, in preclinical pharmaceutical research, cytotoxicity assays are used to screen potential drug candidates and to identify compounds that may have adverse effects on cells, allowing researchers to prioritize safer and more promising candidates for further drug development. An important aspect to consider is that the effects of drugs on proliferation or apoptosis are highly dependent on cell physiology, which varies between different cell types, cell growth stages, health, genetics, and microenvironments. Due to this complexity, cytotoxicity studies should consider the specific characteristics of the cells, carefully designing experiments to account for these factors.

Recently, the results of two large-scale pharmacogenomic studies were compared. These studies encompassed over 450 cancer cell lines and evaluated various parameters such as gene expression, copy number, genome sequence, and pharmacological response to approximately 15 anticancer drugs. Notably, despite the use of common estimators, the pharmacologic drug response showed a high discordance between the studies [11,12]. These comparisons suggested a lack of appropriate standardization of laboratory and assay protocols to study drug responses in pharmacogenomic experiments. Poorly optimized protocols can lead to unreliable and inconclusive results, hindering progress in understanding how various substances affect cell viability and health. Such variability not only makes it challenging to compare results between studies but also raises questions about the reliability of findings, potentially leading to misguided conclusions about the safety and efficacy of substances under investigation.

Similarly, for the resazurin-based viability assay, the cell-specific dehydrogenase and reductase activity can affect the results. Additionally, there are numerous other variables to consider, such as the excitation (Ex) and emission (Em) wavelengths (λ) utilized for FI measurement, the incubation time, and the cell concentration and type.

To enhance the credibility and relevance of cytotoxicity studies, there is an urgent need for standardized and rigorously optimized laboratory protocols, ensuring consistent and accurate results and ultimately advancing drug development and safety assessments. To address this issue, the scientific community has invested significant efforts in developing ISO standards [13,14], guidelines [3,15,16], and standard operating procedures (SOPs) [17] for selecting and utilizing cytotoxicity assays effectively. Many of these standards and guidelines are generic and lack detailed coverage of all essential parameters for effective standardization. Recently, some researches have focused on a more detailed investigation of the specific aspects to be optimized in resazurin-based viability assays for particular cell types. In 2019, Rezende et al. conducted an analysis and validation of both the analytical and biological parameters of the resazurin-based assay for assessing cell viability and cytotoxicity in oral squamous carcinoma and glioblastoma cells. This research aimed to enhance the transition from in vitro to in vivo studies [18]. In 2020, Larsson et al. took an important step by demonstrating that identifying potential confounders and subsequently optimizing cell line-specific parameters could enhance the repeatability and reproducibility of cell-based drug screens [19]. Furthermore, in 2021, an ASTM standard practice was published detailing the procedures for conducting a nondestructive proliferation test for mammalian cells based on the resazurin viability test on 3D scaffolds [20].

Even if an SOP describing the rationale and methodology for optimizing cytotoxicity experiments using the resazurin-based viability assay would be highly beneficial, as of now, such comprehensive guidelines are not available. SOPs play a crucial role in cell biology, as they provide a standardized set of guidelines and protocols for conducting experiments to ensure the consistency, accuracy, and reproducibility in results. Establishing and following an SOP for cell viability assays is indispensable for minimizing variability between experiments, making it easier to compare results across different studies and laboratories [21,22].

This manuscript, with the relative SOP Supplementary File (SOP S1), contributes to the field by describing an SOP aimed at optimizing resazurin-based viability assays in terms of the selection of the best Ex and Em wavelengths, the selection of appropriate incubation times, a definition of assay limits, and an evaluation of repeatability, reproducibility, and measurement uncertainty. The optimization has the ultimate goal of improving the reliability of cytotoxicity tests.

The SOP Supplementary File describes in detail the protocols to perform the experimental workflow; in addition, it reports several recommendations, including suggestions on control samples, and for measuring cell growth and cytotoxicity employing the resazurin assay.

## 2. Optimization Workflow

### 2.1. Excitation and Emission Wavelengths

The selection of optimal Ex and Em wavelengths for resorufin fluorescence measurements is essential for obtaining accurate, sensitive, and reproducible data while minimizing background interference. While resorufin itself remains consistent regardless of the cell type, the cellular environment can significantly influence its excitation and emission properties. For instance, autofluorescence originating from cellular components may overlap with the emission spectrum of the fluorophore. Moreover, variations in intracellular components, such as proteins, lipids, and nucleic acids, can alter the local microenvironment. Additionally, differences in pH levels across cell types and media can impact the resorufin fluorescence intensity and spectral profile.

While resorufin, produced by the enzymatic conversion of resazurin, is a highly fluorescent molecule, resazurin itself has relatively low fluorescence within the visible spectrum [23]. Data from the literature give rather variable indications for the optimal λ_Ex_ and λ_Em_ for resorufin, with suggested values spanning from 530 nm to 570 nm for Ex and from 580 nm to 620 nm for Em.

To select the best combinations of λ_Ex_ and λ_Em_ for a specific cell type at a given concentration of resazurin, an evaluation of different combinations falling within the resazurin fluorescence spectra was necessary. The optimal combination will achieve the highest fluorescence intensity while minimizing the background signal. The approach used to achieve the best signal-to-noise ratio and accuracy is detailed in the SOP S1 file (page 5).

### 2.2. Incubation Time

The recommended incubation time for a resazurin assay typically ranges from 30 min to 4 h. Selecting the correct incubation time, strictly dependent on the cell type and cell confluency, is a critical point for obtaining accurate, comparable, and reliable results. Indeed, a prolonged incubation time when the cell concentration is high can lead to the depletion of the cells’ available resazurin, affecting the direct correlation between its reduction and the number of viable cells. In contrast, a too short incubation time does not allow for an accurate measure of the cells’ proliferative activity when their number is low [9]. Furthermore, the rate of resazurin reduction is influenced by the metabolic rate of the cells, and different cell types and experimental conditions can show different metabolic kinetics. These important limitations are often overlooked. The SOP S1 file (page 8) describes the steps needed for the process of optimizing the incubation time for a specific cell type at a given resazurin concentration, taking into account the cell confluency.

### 2.3. Limit of Blank, Limit of Detection, and Limit of Quantification

Characterizing the analytical performance of a laboratory test is critical to understand its capabilities and limitations, and ensuring that it is “fit for purpose”.

The Limit of Blank (LoB) is defined as “the highest apparent analyte concentration expected to be found when replicates of a sample containing no analyte are tested” [24]. Several replicates, usually from 10 to 20, of blank (no-cell control) have to be measured to estimate the LoB by calculating the mean and the standard deviation (SD). The formula to estimate the LoB is reported below [25]:

(1) $$LOB=mean_{Blank}+1.645(SD_{Blank})$$

The factor 1.645 corresponds to the critical value for a one-sided 95% confidence interval (CI) in a normal distribution.

While the LoB is conceptualized as a concentration, its determination in the context of the resazurin assay involves the measurement of fluorescence intensity (FI). The formula mentioned above serves to establish a threshold value, beyond which signals are judged to be significantly different from background noise (LoB_FI_). Since FI is directly proportional to the concentration of the analyte (viable cells), determining the LoB as a concentration needs the generation of a cell calibration curve. Applying a regression analysis relating FI values to known cell concentrations allows for the interpolation of the LoB_FI_ value calculated using the aforementioned formula (1). Consequently, the LoB value can be obtained as a concentration (SOP S1 file, page 10).

The Limit of Detection (LoD) represents “the lowest amount of an analyte in a sample which can be detected but not necessarily quantitated as an exact value” [26]. The LoD is therefore higher than the LoB [20]. There are different methods for LoD estimation [25,26]. The two most used methods are listed below:

- Starting from both the LoB and replicates of a known sample at a low analyte concentration, by using the following formula [24]:
  (2) $$LoD=mean_{Blank}+1.645\left(SD_{Low\;concentration\;sample}\right)$$

Assuming a Gaussian distribution (one-side) of the low concentration samples, approximately 95% of the values are expected to exceed the previously estimated LoB. Conversely, approximately 5% of the low concentration samples may yield values below the LoB, potentially leading to an erroneous interpretation that no analyte is present [24].

- By using a linear calibration curve (recommended), the LoD can be expressed as
  (3) $$LoD=\frac{3.3\left(SD_{response}\right)}{S}$$
  where *SD_response_* is the SD of either the *y*-intercept of the regression line (recommended), regression line, or the blank; and *S* = the slope of the calibration curve. The factor 3.3 in the formula above corresponds to the critical value for a one-sided 99% CI in a normal distribution and it is used to approximate a 99.7% CI.

The Limit of Quantification (LoQ) is the lowest concentration at which the analyte can be detected with acceptable precision and accuracy [27]. The LoQ can be equal to or greater than the LoD. The LoQ can be approximated by using a linear calibration curve [25,26] as follows:

(4) $$LoQ=\frac{10\left(SD_{response}\right)}{S}$$

where *SD_response_* is the SD of either the *y*-intercept of the regression line (recommended), regression line, or the blank; and *S* = the slope of the calibration curve. The use of a factor of 10 is a widely accepted practice, based on statistical considerations that aim to provide a sufficient separation between the signal associated with the analyte and the background noise. In addition, in a normal distribution, approximately 99.7% of the data fall within three SDs of the mean. By using a factor of 10, there is a high probability that the measured value is well above the noise level.

It is advisable to evaluate more than one calibration curve, performing the experiments on different days. The results can change depending on which method is used to determine the LoD and the LoQ. Regardless of the technique chosen, an adequate number of samples prepared at or near the LoD and the LoQ should be analyzed to demonstrate the appropriateness of the estimated values as part of the validation process.

The steps outlining the process for estimating and validating the LoB, the LoD, and the LoQ using the calibration curve method are reported in the SOP S1 file (page 10).

### 2.4. Repeatability, Reproducibility, and Measurement Uncertainty

Repeatability and reproducibility are ways of measuring the precision of a test. Repeatability, defined as “measurement precision under a set of repeatability conditions of measurement” [28], assesses the consistency of measurements obtained using a single instrument or operator under identical conditions; reproducibility, defined as “measurement precision under reproducibility conditions of measurement” [28], evaluates the agreement between the measurements conducted under nonhomogeneous conditions. Regarding resazurin viability tests, experimental repeatability can be determined as the SD of multiple repeated measurements of the same sample acquired under identical conditions [8,29]. Reproducibility can be calculated as the SD of multiple independent measurements of the same sample conducted under varying conditions (e.g., different days and/or operators) [8,29]. Both repeatability and reproducibility contribute to the assessment of measurement uncertainty (MU), a critical factor in ensuring the reliability of test results. MU is defined as a “nonnegative parameter characterizing the dispersion of the quantity values being attributed to a measurand, based on the information used” [28]. It is frequently indicated as the measured value ± expanded uncertainty (*U*), representing the interval in which the result is expected with a certain degree of confidence. To evaluate the MU, the contributions of the main sources of uncertainty should be included [30,31]. In the specific case of resazurin assays, repeatability, reproducibility, and uncertainty related to the pipette used to transfer metabolized resazurin in the plate for FI measurement should be considered [8,29]. Although a lower MU corresponds to greater precision, there is no fixed value for the MU that is considered to be a “good” value. Considering the purpose of your measurement, analyzing data from the literature, and referring to standards and stakeholders are useful to define acceptable limits or tolerances for a specific experiment. The recommended protocol to evaluate the repeatability, reproducibility, and MU for the resazurin assay is provided in the SOP S1 file (page 14).

## 3. Experimental Approach

Resazurin at a working solution (WS) concentration is generally nontoxic to cells, allowing time-lapse experiments. After the incubation step, transferring resazurin WS to a new plate for FI measurement is recommended but not mandatory. This implies that FI can be measured directly in the plate where the cells are seeded. In this case, it is necessary to use a plate suitable for both cell culture and fluorescence measurement (e.g., a plate with black/white walls and a transparent glass bottom). If you choose this approach, it is essential to include two blank samples in your experiments:

- “No-Cell control” (Blank 1), with resazurin WS only;
- “No-Resazurin WS” (Blank 2), with cells in the culture medium without resazurin WS.

Both the FI values for Blank 1 and 2 should be subtracted from the FI value of the unknown samples.

While this approach has advantages in terms of the number of wells/plasticware used, especially during the assay optimization steps, it has some disadvantages, including potential cell interference in the well (which can vary based on cell confluence) and the risk of losing sterility if the instrument (i.e., a spectrophotometer such as microplate readers) does not allow an FI measurement with the lid on. Based on this information, you should select the most appropriate experimental strategy and optimize the assay using the same approach you intend to use for experimental measurements.

## 4. Applications

Cell viability and proliferation assays play a crucial role in drug discovery for investigating growth factors, cytokines, and cytotoxic substances. These assays also find extensive use in various biomedical applications, including in vitro differentiation studies and the assessment of cell colonization within 3D scaffolds, where an appropriate cell number is a key factor in achieving the desired results. Furthermore, the possibility of monitoring the same sample over time without altering the system is essential for studying cellular behavior and functions [8] (see SOP S1 file, page 16, for assay setup recommendations).

### 4.1. Relative Quantification

For proliferation assays, the results are usually expressed as the fold change (FC, a measure that describes how much a quantity changes between an original and a subsequent measurement [28]), normalizing results on untreated cells. For cytotoxicity tests, the results are usually expressed as percentages, normalized to positive and negative control samples, by using the “min-max normalization” method (see SOP S1 file, page 17, for further details).

### 4.2. Absolute Quantification

Since there is a direct correlation between resorufin FI and the number of proliferating cells, it is possible to extrapolate the number of viable cells in a sample using a calibration curve. A calibration curve must be used in each independent experiment and should be incubated with resazurin WS under identical conditions as the unknown samples (see SOP S1 file, page 18, for further details).

## 5. Outlier Management and Significance Assessment

### 5.1. Outliers

By carrying out all the steps described above, the possibility of obtaining outlier data is significantly reduced. However, the risk of addressing technical errors cannot be completely eliminated. In a cell viability assay, the presence of outlier data warrants careful consideration to ensure the accuracy and reliability of results. Outliers, defined as “a result that is very different from the main group of results” [32], may arise from various sources, including technical errors or biological variability. The proper handling of outliers involves assessing their impact on the overall dataset. Depending on the context, outliers may be retained, adjusted, or excluded after a thorough examination. Statistical methods, graphical representations, and an understanding of the biological system’s variability are crucial in making informed decisions about the treatment of outlier data. Managing outliers appropriately contributes to the robustness and validity of cell viability assay results, enhancing the overall interpretation of experimental outcomes (see SOP S1 file, page 20).

### 5.2. Statistical Differences between Populations

When a statistically significant difference is observed between two populations in a study and the calculated difference lies within the *U* of the measurement method, caution is needed in drawing definitive conclusions. Indeed, this condition suggests that the apparent statistical significance may be compromised by the variability and potential errors associated with the measurement.

Additionally, while a statistical difference might be considered significant, it is crucial to assess its biological significance and relevance. It is essential to discern whether the identified statistical significance holds biological implications.

These assessments ensure that research findings are not only statistically robust but also have real-world and impactful relevance (see SOP S1 file, page 20).

## 6. Discussion

Viability tests play a crucial role in both fundamental and translational research, particularly in the field of cell-based pharmacogenomics for preclinical drug screening. These tests are pivotal in determining the viability and health of cells subjected to various experimental conditions, providing crucial insights into their response to potential therapeutic agents. Unfortunately, these studies often encounter challenges related to low inter-laboratory reproducibility, which ultimately leads to unreliable data [11,33,34]. As a result, the credibility and translational potential of preclinical drug screening efforts are often compromised, hindering the advancement of therapeutic development initiatives. In recent years, an increasingly high and worrying number of irreproducible preclinical studies has emerged. It is estimated that laboratory protocols are one of the primary causes of irreproducibility in 10.8% of cases [12], highlighting the critical role that procedural inconsistencies and methodological variability play in undermining research outcomes. Consequently, improving the reliability of results is a major challenge in life sciences. Consistent and reliable results are essential not only for advancing scientific knowledge but also for facilitating the translation of research findings into clinical applications. Regardless of the specific method used to determine the cellular response to treatment, the results obtained should be consistent. To address this challenge, one effective strategy involves optimizing experimental parameters. Indeed, by carefully fine-tuning variables such as the concentration, volume, and incubation time, it is possible to minimize sources of variability and enhance the reproducibility of the findings. SOPs are essential requirements in clinical laboratories, serving as a key component of a quality management system and playing a pivotal role in guaranteeing the reliability, precision, and uniformity of results [21,22]. By delineating precise steps and guidelines for each aspect of an experiment, SOPs help ensure the consistency of results across different laboratories and experimental conditions.

In this manuscript, we presented a comprehensive optimization approach (namely an SOP) applicable to the resazurin assay across various cell lines.

Our primary focus was to optimize critical experimental parameters, such as the concentration, volume, and incubation time. By systematically optimizing these parameters, we aimed to maximize the reliability of the resazurin assay across various cell lines, ensuring consistent and reproducible results under different experimental conditions.

Additionally, we placed a significant emphasis on the rigorous assessment of data quality throughout the experimental process, to enhance the robustness and reliability of our results, minimizing potential sources of variability and error. A key aspect of our approach involved minimizing the MU, while also characterizing and understanding the confidence limits of the viability test.

By carefully optimizing the experimental parameters and implementing standardized protocols, researchers can significantly enhance the robustness and reliability of viability testing results. This not only fosters greater confidence in research findings but also promotes the reproducibility and translatability of preclinical studies, ultimately advancing scientific knowledge and contributing to the development of effective therapeutic interventions.

## 7. Conclusions

In conclusion, the presented SOP offers a systematic approach to optimize the resazurin assay, addressing challenges of variability and enhancing reliability. By adhering to standardized protocols and correct parameter optimization, researchers can ensure consistent and reproducible results, advancing the reliability of viability testing in preclinical drug screening.

## Data Availability

No new data were created or analyzed in this study. Data sharing is not applicable to this article.

## Supplementary Materials

The following supporting information can be downloaded at: https://www.mdpi.com/article/10.3390/bios14040156/s1, SOP S1: Standard operating procedure to optimize resazurin-based viability assays [20,35,36,37,38,39,40,41,42].

## References

[1] Kuete V., Karaosmanoğlu O., Sivas H. (2017). Anticancer Activities of African Medicinal Spices and Vegetables. Medicinal Spices and Vegetables from Africa.

[2] O’Brien J., Wilson I., Orton T., Pognan F. (2000). Investigation of the Alamar Blue (Resazurin) Fluorescent Dye for the Assessment of Mammalian Cell Cytotoxicity: Resazurin as a Cytotoxicity Assay. Eur. J. Biochem..

[3] Riss T.L., Moravec R.A., Niles A.L., Duellman S., Benink H.A., Worzella T.J., Minor L., Markossian S., Grossman A., Brimacombe K., Arkin M., Auld D., Austin C., Baell J., Chung T.D.Y., Coussens N.P., Dahlin J.L. (2004). Cell Viability Assays. Assay Guidance Manual.

[4] Stockert J.C., Horobin R.W., Colombo L.L., Blázquez-Castro A. (2018). Tetrazolium Salts and Formazan Products in Cell Biology: Viability Assessment, Fluorescence Imaging, and Labeling Perspectives. Acta Histochem..

[5] Vakifahmetoglu-Norberg H., Ouchida A.T., Norberg E. (2017). The Role of Mitochondria in Metabolism and Cell Death. Biochem. Biophys. Res. Commun..

[6] Penner M.H., Nielsen S.S. (2017). Ultraviolet, Visible, and Fluorescence Spectroscopy. Food Analysis.

[7] Neufeld B.H., Tapia J.B., Lutzke A., Reynolds M.M. (2018). Small Molecule Interferences in Resazurin and MTT-Based Metabolic Assays in the Absence of Cells. Anal. Chem..

[8] Divieto C., Sassi M.P. (2015). A First Approach to Evaluate the Cell Dose in Highly Porous Scaffolds by Using a Nondestructive Metabolic Method. Future Sci. OA.

[9] Gong X., Liang Z., Yang Y., Liu H., Ji J., Fan Y. (2020). A Resazurin-Based, Nondestructive Assay for Monitoring Cell Proliferation during a Scaffold-Based 3D Culture Process. Regen. Biomater..

[10] Uzarski J.S., DiVito M.D., Wertheim J.A., Miller W.M. (2017). Essential Design Considerations for the Resazurin Reduction Assay to Noninvasively Quantify Cell Expansion within Perfused Extracellular Matrix Scaffolds. Biomaterials.

[11] Haibe-Kains B., El-Hachem N., Birkbak N.J., Jin A.C., Beck A.H., Aerts H.J.W.L., Quackenbush J. (2013). Inconsistency in Large Pharmacogenomic Studies. Nature.

[12] Freedman L.P., Cockburn I.M., Simcoe T.S. (2015). The Economics of Reproducibility in Preclinical Research. PLoS Biol..

[13] (2009). Biological Evaluation of Medical Devices—Part 5: Tests for In Vitro Cytotoxicity.

[14] (2018). Nanotechnologies—In Vitro MTS Assay for Measuring the Cytotoxic Effect of Nanoparticles.

[15] Kamiloglu S., Sari G., Ozdal T., Capanoglu E. (2020). Guidelines for Cell Viability Assays. Food Front..

[16] Präbst K., Engelhardt H., Ringgeler S., Hübner H., Gilbert D.F., Friedrich O. (2017). Basic Colorimetric Proliferation Assays: MTT, WST, and Resazurin. Cell Viability Assays.

[17] Zhang N., Kral V., Zoschke C., Gerecke C., Schäfer-Korting M., Department of Biology, Chemistry, Pharmacy Institute for Pharmacy (Pharmacology and Toxicology) Königin-Luise-Str. 2+4 14195 Berlin Germany Viability Assessment of Cell Monolayers with MTT Reduction Assay in 96-Well Plates 2017; Standard Operating Procedure (SOP). https://www.bb3r.de/forschungsplattform/Standardarbeitsvorschriften/SOP_cell-viability_170116.pdf.

[18] Rezende N., Ceron Jayme C., Brassesco M.S., Claudio Tedesco A., De Oliveira H.F. (2019). Standardization of a Resazurin-Based Assay for the Evaluation of Metabolic Activity in Oral Squamous Carcinoma and Glioblastoma Cells. Photodiagnosis Photodyn. Ther..

[19] Larsson P., Engqvist H., Biermann J., Werner Rönnerman E., Forssell-Aronsson E., Kovács A., Karlsson P., Helou K., Parris T.Z. (2020). Optimization of Cell Viability Assays to Improve Replicability and Reproducibility of Cancer Drug Sensitivity Screens. Sci. Rep..

[20] (2021). 21_Standard Practice for Quantifying Cell Proliferation in 3D Scaffolds by a Nondestructive Method.

[21] Hollmann S., Frohme M., Endrullat C., Kremer A., D’Elia D., Regierer B., Nechyporenko A., on behalf of Cost Action CA15110 (2020). Ten Simple Rules on How to Write a Standard Operating Procedure. PLoS Comput. Biol..

[22] Freeman K.P., Cook J.R., Hooijberg E.H. (2021). Standard Operating Procedures. Javma.

[23] Csepregi R., Lemli B., Kunsági-Máté S., Szente L., Kőszegi T., Németi B., Poór M. (2018). Complex Formation of Resorufin and Resazurin with Β-Cyclodextrins: Can Cyclodextrins Interfere with a Resazurin Cell Viability Assay?. Molecules.

[24] CLSI (2012). Evaluation of Detection Capability for Clinical Laboratory Measurement Procedures.

[25] Chandran S., Singh R.S.P. (2007). Comparison of Various International Guidelines for Analytical Method Validation. Pharmazie.

[26] Committee for Medicinal Products for Human Use ICH Guideline Q2(R2) on Validation of Analytical Procedures 2022; ICH Guideline Q2(R2) on Validation of Analytical Procedures; Guideline. https://www.ema.europa.eu/en/documents/scientific-guideline/ich-guideline-q2r2-validation-analytical-procedures-step-2b_en.pdf;.

[27] Hay I.D., Bayer M.F., Kaplan M.M., Klee G.G., Larsen P.R., Spencer C.A. (1991). American Thyroid Association Assessment of Current Free Thyroid Hormone and Thyrotropin Measurements and Guidelines for Future Clinical Assays. The Committee on Nomenclature of the American Thyroid Association. Clin. Chem..

[28] JCGM Member Organizations (2012). International Vocabulary of Metrology—Basic and General Concepts and Associated Terms (VIM).

[29] Divieto C., Revel L., Sassi G., Sassi M.P. (2013). Uncertainty Analysis of Cell Counting by Metabolic Assays. J. Phys. Conf. Ser..

[30] Milinković N., Ignjatović S., Šumarac Z., Majkić-Singh N. (2018). Uncertainty of Measurement in Laboratory Medicine. J. Med. Biochem..

[31] (2008). Jcgm, JCGM Evaluation of Measurement Data—Guide to the Expression of Uncertainty in Measurement. Int. Organ. Stand. Geneva ISBN.

[32] Borowski E.J., Borwein J.M. (2012). Collins Dictionary of Mathematics.

[33] Prinz F., Schlange T., Asadullah K. (2011). Believe It or Not: How Much Can We Rely on Published Data on Potential Drug Targets?. Nat. Rev. Drug. Discov..

[34] Horvath P., Aulner N., Bickle M., Davies A.M., Nery E.D., Ebner D., Montoya M.C., Östling P., Pietiäinen V., Price L.S. (2016). Screening out Irrelevant Cell-Based Models of Disease. Nat. Rev. Drug. Discov..

[35] Lavogina D., Lust H., Tahk M.-J., Laasfeld T., Vellama H., Nasirova N., Vardja M., Eskla K.-L., Salumets A., Rinken A. (2022). Revisiting the Resazurin-Based Sensing of Cellular Viability: Widening the Application Horizon. Biosensors.

[36] Aronhime S., Calcagno C., Jajamovich G.H., Dyvorne H.A., Robson P., Dieterich D., Isabel Fiel M., Martel-Laferriere V., Chatterji M., Rusinek H. (2014). DCE-MRI of the Liver: Effect of Linear and Nonlinear Conversions on Hepatic Perfusion Quantification and Reproducibility. Magn. Reson. Imaging.

[37] Beah A., Kamara A.Y., Jibrin J.M., Akinseye F.M., Tofa A.I., Adam A.M. (2020). Simulating the Response of Drought–Tolerant Maize Varieties to Nitrogen Application in Contrasting Environments in the Nigeria Savannas Using the APSIM Model. Agronomy.

[38] Revel L., Santiano M. (2022). RT 15/2022 Manual Micropipettes: Internal Procedure for Periodic Verification, Data Acquisition and Processing Software.

[39] (2022). Piston-Operated Volumetric Apparatus—Part 2: Pipettes.

[40] (2022). Piston-Operated Volumetric Apparatus—Par 6: Gravimetric Reference Measurement Procedure for the Determination of Volume.

[41] Working Group 1 of the Joint, Committee for Guides in Metrology (JCGM/WG 1) JCGM 100:2008 Evaluation of Measurement Data—Guide to the Expression of Uncertainty in Measurement 2008. https://ncc.nesdis.noaa.gov/documents/documentation/JCGM_100_2008_E.pdf.

[42] Barwick V. (2003). Preparation of Calibration Curves—A Guide to Best Practice.

