# Supplementary material for: Standard Operating Procedure to Optimize Resazurin-Based Viability Assays
Source: Biosensors (Basel). 2024 Mar 26;14(4):156. doi: 10.3390/bios14040156 (PMC11048620; doi:10.3390/bios14040156)
Supplement: Supplementary file 1 [file biosensors-14-00156-s001.zip › biosensors-2903885-supplementary.pdf]

SOP

Standard operating procedure to optimize resazurin-based viability assays

<table>
<tr><td colspan="3">Institution

Istituto Nazionale di Ricerca Metrologica – INRiM, Turin, Italy</td><td>INRiM ISTITUTO NAZIONALE DI RICERCA METROLOGICA</td></tr>
<tr><td colspan="3">Title

Standard operating procedure to optimize resazurin-based viability assays</td><td>Date

2023-10-27</td></tr>
<tr><td>Document No.

INRIM_SOP Bio_001</td><td>First edition

2023-10-27</td><td colspan="2">Version

1</td></tr>
<tr><td>Issued by

J. Petiti</td><td>Reviewed by

C. Divieto, L. Revel</td><td colspan="2">Approved by

C. Divieto</td></tr>
<tr><td colspan="4">Scope

Optimization of resazurin-based viability assays to improve the reliability of cytotoxicity data</td></tr>
</table>

Index

Introduction 3

Aims 3

Terminology 3

List of abbreviations 3

Materials 4

Equipment *4*

Consumables *4*

Reagents *4*

General information 4

Experimental workflow 5

Selection of excitation and emission wavelengths *5*

Identification of optimal incubation time *8*

Estimation of Limit of Blank (LoB), Limit of Detection (LoD), and Limit of Quantification (LoQ) *10*

Validation of LoD and LoQ *12*

Assessment of repeatability, reproducibility, and measurement uncertainty *14*

Assays setup 16

Experiment setup *16*

Results quantification 17

Relative quantification *17*

Absolute quantification *18*

Outlier management and significance assessment 20

Outliers *20*

Statistically differences between data populations *20*

References 21

Introduction

The resazurin-based viability assay is a widely used method for assessing the viability and metabolic activity of cells, commonly employed in cell biology and drug testing. It relies on the reduction of resazurin, a non-fluorescent blue dye, to resorufin, a highly fluorescent pink compound, by living cells. Resazurin-based viability assay can be applied to various cell types and experimental conditions to evaluate cell growth, cytotoxicity, and drug sensitivity. The assay provides a convenient and quantitative method to assess the vitality of cells, making it a fundamental tool in cell biology and drug development research. While the resazurin-based viability test is a widely used method, the results reliability can be affected by various factors, such as cells metabolic characteristics, environmental and experimental conditions. Improving the reproducibility of results has recently emerged as one of the major challenges in laboratory medicine.

Aims

This SOP describes how to optimize the critical experimental parameters of resazurin-based viability assays, including excitation (Ex) and emission (Em) wavelengths (λ) and incubation times. It also provides recommendations for determining assay limits, such as the Limit of Blank (LoB), Limit of Detection (LoD), and Limit of Quantification (LoQ). Additionally, it details how to assess results accuracy, consistency, and quality through repeatability, reproducibility, and measurement uncertainty, all with the ultimate goal of improving the reliability of cytotoxicity test results. This SOP is limited to the evaluation of immobilized cells, which can include cell adhesion to a culture dish or a scaffold, or cell encapsulation within a hydrogel. Furthermore, it is applicable exclusively to cells able to metabolize resazurin, namely only to living/metabolically active cells.

Terminology

In the following SOP, the term “must” is used to denote mandatory steps or requirements within the protocol. When "must" is employed, adherence is non-negotiable, as these actions are essential for the correct execution and reliability of the procedure. Conversely, the term "should" has been employed to highlight recommended practices that, while not mandatory, are strongly advised for achieving optimal results.

In the absence of explicit definitions in the SOP, refer to the main text for clarification and definitions.

List of abbreviations

a.u., arbitrary units; CV, coefficient of variation; Em, emission; Ex, excitation; FC, fold change; FI, fluorescence intensity; h, hours; LoB, Limit of Blank; LoD, Limit of Detection; LoQ, Limit of Quantification; RT, room temperature; SD, standard deviation; SE, standard error; SOP, standard operating procedure; *u*, combined measurement uncertainty; *U*, expanded uncertainty; WS, working solution; λ, wavelengths.

Materials

Equipment

- Standard equipment for cell culture (e.g., centrifuge for tubes, cell incubator, laminar flow hood, optical microscope, micropipettes, etc.)
- Cell counting chamber or automatic cell counter
- Fluorescence reader

Consumables

- Cell culture plates
- 96-well plate for fluorescence measurements (e.g., black plates)
- Centrifuge tubes (15 and 50 mL)
- Microtubes (1.5 and 2 mL)
- Pipette tips
- Serological Pipettes

Reagents

- Cell culture medium (specific for the cell line/s of interest)
- Cell culture medium supplements to support cell growth and viability (specific for the cell line/s of interest)
- Trypsin or equivalent
- milliQ-H_2O
- Resazurin sodium salt

General information

Unless stated otherwise, all procedures should be performed under sterile laminar flow conditions. Protective gloves should be worn and all work areas should be thoroughly cleaned before and after testing.

Experimental workflow

The steps outlined below must be executed in the sequence specified by this SOP, but they do not need to be completed on consecutive days.

Before starting:

Dissolve resazurin powder into Mill-Q H_2O to an appropriate concentration, sterilize by filtration through a 0.22 um filter, and prepare aliquots to store at -20°C.

Selection of excitation and emission wavelengths

Step 1:

1. Detach cells following the manufacturer's suggestions;
2. Resuspend cells in an appropriate volume of medium and count them;
3. Establish at least 3 different cell concentrations representing low, medium, and high confluency (e.g., 10%, 50%, and 90% cell confluency[1]);
4. Seed the appropriate cell number in a multi-well plate (at least triplicate for each condition is recommended[2]). If possible, consider using a cell culture medium with low FBS concentration (e.g., 1-2% FBS) to avoid excessive cell growth. Consider the number of technical replicates and the selected λ_{Ex}-λ_{Em} values for each level of cell confluency. For instance, if you have technical triplicates and 6 λ_{Ex}-λ_{Em} combinations, you will require 18 wells for each level of cell confluency, including the Blank (= absence of cells, i.e. "No-Cell Control");
5. Culture cells until they are firmly attached to the bottom of the plate using standard cell culture conditions (recommended time: 12-18 hours (h)). Figure 1 illustrates an example of an experiment scheme.

[1] Cell confluency is the degree to which the surface of a cell-culture dish is covered by adherent cells [1].

[2] Replicates are essential for evaluating experimental variation. Considering the variability in cultured cells, it is advisable to include a sufficient number of replicates to fulfill the statistical prerequisites of the experiment. It is recommended to have a minimum of three biological replicates for each condition and to perform the entire assay three times [2].

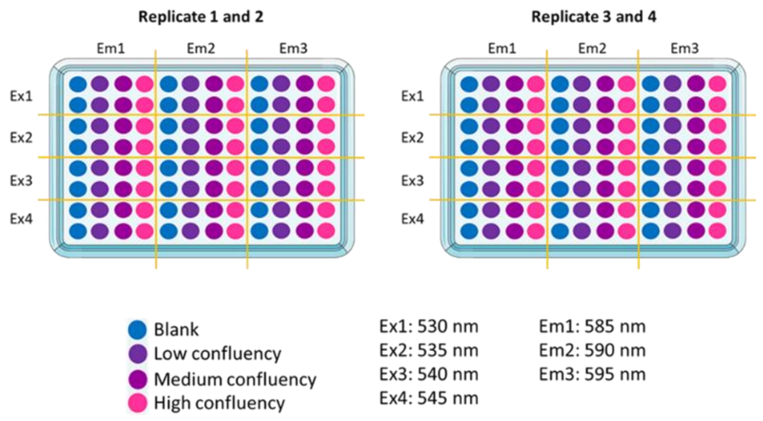

Figure S1. Example of an experimental plate scheme to evaluate the best λ_{Ex}-λ_{Em} combination. Blank represents the "No-Cell Control"

Step 2:

6. Allow the resazurin reach to room temperature (RT). A 37°C water bath may be used to eventually thaw the reagent. Protect the resazurin from direct light;
7. Prepare the resazurin Working Solution (WS) with a concentration ranging between 40 and 50 µM by diluting resazurin-H_2O solution in the complete cell culture medium, and warm it to 37°C until use. This concentration range is generally suitable for most cells. However, it is possible to adjust it, taking into account that low concentrations (≤25 µM) might lead to resazurin depletion with highly confluent cells, while higher concentrations (≥100 µM) of resazurin could potentially be cytotoxic [3];
8. Remove the assay plate from the incubator and gently eliminate the medium from the wells;
9. Add an appropriate volume of resazurin WS in each well. Add resazurin WS in one or more empty wells to set up the "No-Cell Control" (Blank). The volume of resazurin WS depends on the multi-well plate used. For this experiment, a 96-well plate is recommended. Refer to Table 1 for selecting the correct resazurin WS volume[3];

Culture plate	Surface area [cm^2]	Resazurin WS volume [mL]
96-well	0.32	0.1
48-well	1.1	0.35
24-well	1.9	0.6
12-well	3.5	1.1
6-well	9.6	3

Table S1. Resazurin WS volumes recommended for different multi-well plates in order to maintain the scalability ratio.

[3] In line with the recommended volume of 100 uL/well for the 96-well plate, the volumes suggested for the other plates are proportionally adjusted based on their well surface area. This ensures that, regardless of the surface, cells may receive and metabolize an equivalent amount of Resazurin. Consequently, this approach guarantees the comparability of results obtained across different plates.

10. Incubate cells using standard culture conditions for 1–4 h;
11. After the incubation, gently remove the resazurin WS from the wells and transfer 100 µL to a 96-well plate for Fluorescence Intensity (FI) measurement[4];
12. In order to identify the optimal excitation and emission wavelengths (λ_{Ex} and λ_{Em}) combinations, use a Fluorescence Microplate Reader to record the FI at different combinations of λ_{Ex} and λ_{Em} in the ranges Ex: 530 nm-570 nm, Em: 580 nm-620 nm;
13. Calculate the FI mean of replicates (FI_{mean}) for each combination (different cell confluences and Blank for each λ_{Ex}-λ_{Em} combination);
14. Calculate the standard deviation (SD) of replicates for each combination (different cell confluences and Blank for each λ_{Ex}-λ_{Em} combination);
15. Subtract the FI_{mean} of the Blank from the FI_{mean} of each experimental well ($FI_{Sample-Blank}$);
16. Calculate the SD ($FI_{Sample-Blank}$) by propagating the error using the formula:

$$SD(FI_{Sample-Blank}) = \sqrt{SD^2_{Sample} + SD^2_{Blank}}$$

17. For each cell confluency, plot $FI_{Sample-Blank}$ versus λ_{Ex}-λ_{Em} combination. An example for your convenience is reported in Fig 2A. Select the λ_{Ex}-λ_{Em} combinations that allow obtaining the greatest $FI_{Sample-Blank}$ difference between the experimental well and the Blank;
18. For each λ_{Ex}-λ_{Em} combination, plot $FI_{Sample-Blank}$ versus cell concentration (cell n°/area [cm^{-2}]). An example for your convenience is reported in Fig. 2B. Assess the linearity of previously selected λ_{Ex}-λ_{Em} combinations (coefficient of determination (R^2) should be higher than 0.97);
19. If more than one λ_{Ex}-λ_{Em} conditions have similar results ($FI_{Sample-Blank}$ and R^2), choose the one that allows obtaining the best outcome at the lowest cell confluency.

[4] Because frequently there is a minimal discrepancy between the volume dispensed into the well and the volume that can be withdrawn from the well, when using a 96-well plate, it is feasible to either increase the volume up to 105 µL for incubation and maintain 100 µL for FI measurement or to keep the incubation volume at 100 µL and reduce the measurement volume up to 95 µL. In either case, it is essential to ensure consistency by maintaining the same volume of resazurin (for both incubation and fluorescence intensity measurement) across all samples. Applying such a minimal variation (not exceeding 5% of the volume), the amounts of resazurin to be used for other plates with different surface areas can remain as indicated in Table 1.

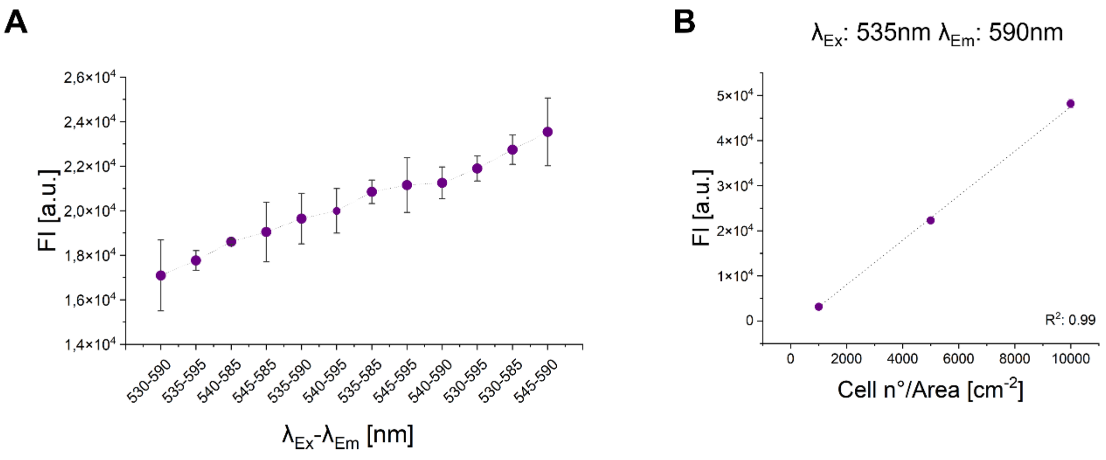

Figure S2. Example of plot to evaluate the best λ_{Ex}-λ_{Em} combination. Error bars indicate the standard deviation (SD). Fluorescence intensity (FI) is expressed as arbitrary units (a.u.). **A)** $FI_{Sample-Blank}$ (*y*-axis) versus λ_{Ex}-λ_{Em} combination (*x*-axis). **B)** $FI_{Sample-Blank}$ (*y*-axis) versus cell concentration (*x*-axis).

Identification of optimal incubation time

<u>Step 3:</u>

1. Detach cells following the manufacturer's suggestions;
2. Resuspend cells in an appropriate volume of medium and count them;
3. Establish at least 5 cells concentration representing very low, low, medium, high, and very high confluency, in the range where there is interested in or it is needed to consider in the experiments;
4. Define different incubation time points to test. Typical resazurin incubation time ranges from 30 min to 4 h. Cells with high growth rates tend to metabolize resazurin rapidly. Refer to literature data to determine suitable incubation times for testing. Start with shorter intervals, such as 15-30 minutes, as certain cell types may quickly reach reaction saturation, and consider expanding them gradually as needed;
5. Seed the appropriate number of cells in a multi-well plate (at least triplicate[2] wells for each confluency condition are recommended). If possible, consider using a cell culture medium with low FBS concentration (e.g., 1-2% FBS) to avoid excessive cells growth. Consider the number of technical replicates and the selected incubation time points for each level of cell confluency. For instance, if you have technical triplicates and 5 incubation time points, you will require 15 wells for each level of cell confluency, including the Blank (= absence of cells, i.e. "No-Cell Control");
6. Culture cells until they are firmly attached to the bottom of the plate using standard cell culture conditions (recommended time: 12-24 h, depending on the cell type). Figure 3 illustrates an example experiment design, with each time point condition evaluated in triplicate.

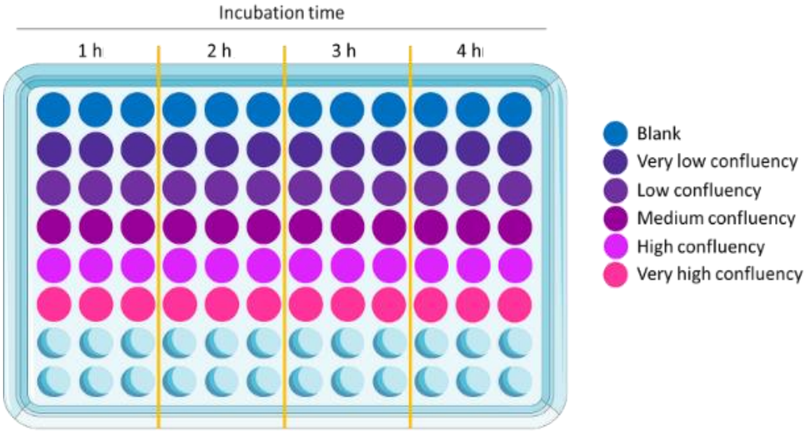

Figure S3. Example of an experimental scheme to evaluate the best incubation time.
Blank represents the "No-Cell Control"

<u>Step 4:</u>

7. Allow the resazurin to reach RT. A 37°C water bath may be used to eventually thaw the reagent. Protect the resazurin from direct light;
8. Prepare the resazurin WS with a concentration ranging between 40 and 50 µM by diluting resazurin-H_2O solution in the complete cell culture medium, and warm it to 37°C until use;
9. Remove the assay plate from the incubator and gently eliminate the medium from the wells;
10. Add an appropriate volume of resazurin WS in each well. Add resazurin WS in one or more empty wells to set up the "No-Cell Control" (Blank). The volume of resazurin WS depends on the multi-well plate used. For this experiment, a 96-well plate is recommended. Refer to Table 1 to select the correct resazurin WS volume;
11. Incubate cells using standard cell culture conditions;
12. At the first defined incubation time (e.g., 1 h), gently remove the resazurin WS from the respective wells and transfer 100 µL in a 96-well plate for FI measurement;
13. Place the plate back into the incubator until the next selected time point is reached;
14. Use a Fluorescence Microplate Reader to record the FI at the previously selected λ_{Ex}-λ_{Em} combination;
15. Repeat steps 12-14 until reaching the last set incubation time point;
16. Calculate the FI_{mean} of replicates for each condition (i.e. different cell concentrations) and consider the specific Blank of each incubation time point;
17. Calculate the SD of replicates for each condition (i.e. different cell concentrations) and Blank samples;
18. For each incubation time evaluated, subtract the FI_{mean} of Blank from the FI_{mean} of all experimental wells ($FI_{Sample\text{-}Blank}$);
19. Calculate the SD ($FI_{Sample\text{-}Blank}$) by propagating the error using the formula:

$$\boldsymbol{SD(FI_{Sample-Blank}) = \sqrt{SD^2_{Sample} + SD^2_{Blank}}}$$

20. For each cell confluency selected, plot $FI_{Sample\text{-}Blank}$ versus incubation time. An example for your convenience is reported in Fig. 4;

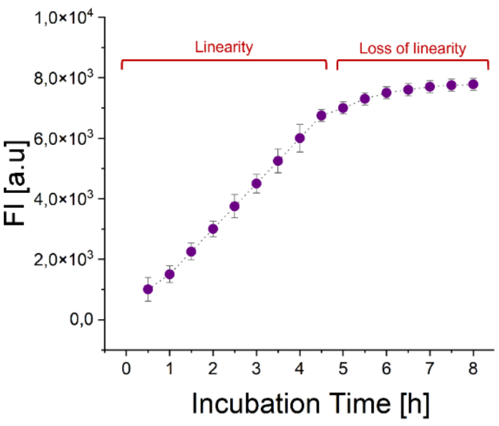

Figure S4. Example of plot to evaluate the optimal incubation time for one of the tested cell confluencies. Error bars indicate the SD. FI is expressed as arbitrary units (a.u.). $FI_{Sample\text{-}Blank}$ (*y*-axis) versus incubation time (*x*-axis).

21. Identify when the linearity of the curve is lost.
22. Using these findings, establish the appropriate incubation time for each upcoming experiment, considering the range of cell concentrations that will be seeded.

Estimation of Limit of Blank (LoB), Limit of Detection (LoD), and Limit of Quantification (LoQ)

The following steps outline the process for estimating LoB, LoD, and LoQ using the calibration curve method.

<u>Calibration curve preparation (Step 5):</u>

1. Detach cells following the manufacturer's suggestions;
2. Resuspend cells in an appropriate volume of medium and count them;
3. Prepare from 5 to 10 cell serial dilutions in a complete culture medium. The standard concentrations should range from very low to low confluency. If possible, consider using a cell culture medium with low FBS concentration (e.g., 1-2% FBS) to avoid excessive cell growth;
4. Seed the cell dilutions in a multi-well plate (at least triplicate[2] wells for each condition are recommended).
5. Culture cells until they are firmly attached to the bottom of the plate using standard cell culture conditions (12-24 h are recommended).

Measurement assessment (Step 6):

6. Allow the resazurin to reach RT. A 37°C water bath may be used to eventually thaw the reagent. Protect the resazurin from direct light;
7. Prepare the resazurin WS with a concentration between 40 and 50 µM in the complete cells culture medium and warm it at 37°C until use;
8. Remove the assay plate from the incubator and gently eliminate the medium from the wells;
9. Add an appropriate volume of resazurin WS in each well. Add resazurin WS in at least 10 empty wells to set up the “No-Cell Control” (Blank). The volume of resazurin WS depends on the multi-well plate used. Refer to Table 1 for selecting the correct resazurin WS volume;
10. Incubate cells using standard conditions;
11. Reached the expected incubation time (previously optimized), gently remove the resazurin WS from the respective wells and transfer 100 µL in a 96-well plate for FI measurement;
12. Use a Fluorescence Microplate Reader to record the FI at the optimal λ_{Ex}-λ_{Em} combination previously selected;
13. Calculate the FI_{mean} and SD of replicates for each test condition (calibration curve and Blank);
14. Plot FI_{mean} versus the concentration of cells seeded (e.g., cell n°/well or cell n°/cm^2) (Fig. 5);

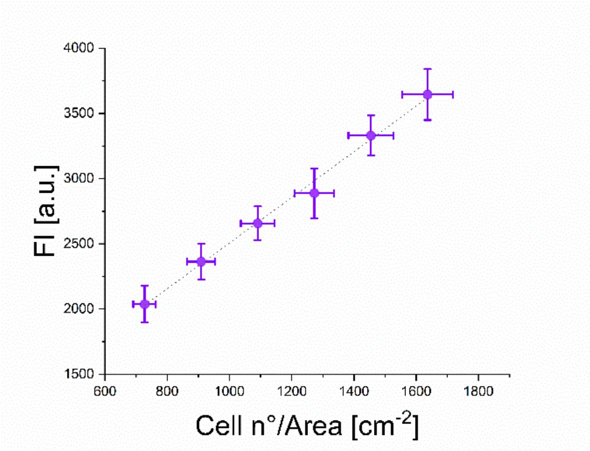

Figure S5. Example of curve plot to evaluate LoB, LoD, and LoQ: $FI_{Sample-Blank}$ (y-axis) versus cell concentration (x-axis). Error bars on y-axis indicate SD, while those on x-axis indicate the uncertainty related to the cell counting[5].

15. Perform a linear regression analysis to obtain the linear regression equation ($y=Sx + b$, where y is the dependent variable (FI), x is the independent variable (concentration of cells), S is the estimated slope, and b is the estimated y-intercept);

[5] Indicating error bars on the nominal value of the cells is recommended, but not mandatory. The error associated with the nominal cell number arises from the variability of the count, calculated on repeated measurements. In our previous studies, the variability associated to the use of Neubauer chamber was estimated to be approximately 5% [4].

16. Ensure that R^2 is higher than 0.97. If R^2 is lower than 0.97, it is possible to exclude one or more points of the curve, keeping at least 5 points;
17. Calculate LoB_{FI} [5,6]:

$$\boldsymbol{LoB_{FI} = mean_{Blank} + 1.645(SD_{Blank})}$$

18. Calculate LoB by interpolating the LoB_{FI} (y value) using the previously derived linear regression equation;
19. Calculate the SD of regression from the Standard Error (SE) of the y-intercept, which is determined as:

$$\boldsymbol{SD_{regression} = SE_{regression} \times \sqrt{n}}$$

20. Calculate LoD and LoQ using the following formula [5,6]:

$$\boldsymbol{LoD = \frac{3.3(SD_{response})}{S}} \qquad \boldsymbol{LoQ = \frac{10(SD_{response})}{S}}$$

$SD_{response}$ = SD of y-intercept of the regression line

S = Slope of the calibration curve

Validation of LoD and LoQ

<u>Step 7:</u>

1. Detach cells following the manufacturer's suggestions;
2. Resuspend cells in an appropriate volume of medium and count them;
3. Prepare two experimental samples, each with a minimum of 10 replicates, at or around the previously estimated LoD and LoQ. If possible, consider using a cell culture medium with low FBS concentration (e.g., 1-2% FBS) to avoid excessive cell growth;
4. Seed the cell dilutions in a multi-well plate;
5. Culture cells until they are firmly attached to the bottom of the plate using standard cell culture conditions (12-24 h are recommended).

<u>Step 8:</u>

6. Allow the resazurin to reach RT. A 37°C water bath may be used to eventually thaw the reagent. Protect the resazurin from direct light;
7. Prepare the resazurin WS with a concentration between 40 and 50 µM in the complete cells culture medium and warm it at 37°C until use;
8. Remove the assay plate from the incubator and gently eliminate the medium from the wells;

9. Add an appropriate volume of resazurin WS in each well. Add resazurin WS in at least 10 empty wells to set up the "No-Cell Control" (Blank). The volume of resazurin WS depends on the multi-well plate used. Refer to Table 1 for selecting the correct resazurin WS volume;
10. Incubate cells using standard conditions;
11. Reached the expected incubation time (previously optimized), gently remove the resazurin WS from the respective wells and transfer 100 µL in a 96-well plate for FI measurement;
12. Use a Fluorescence Microplate Reader to record the FI at the optimal λ_{Ex}-λ_{Em} combination previously selected;
13. Verify the statistical significance of the differences between LoD and LoQ compared to the Blank (e.g., using a t-test). If the differences are not significant, repeat the experiment by increasing the cell concentration for LoD and/or LoQ (Table 2).
14. Calculate the FI_{mean} and SD of replicates for each test condition (LoD, LoQ, and Blank);
15. Subtract the FI_{mean} of Blank from the FI_{mean} of LoD and LoQ ($FI_{Sample\text{-}Blank}$);
16. For both LoD and LoQ, calculate the SD ($FI_{Sample\text{-}Blank}$) by propagating the error using the formula:

$$\boldsymbol{SD(FI_{Sample-Blank})} = \sqrt{\boldsymbol{SD^2_{Sample}} + \boldsymbol{SD^2_{Blank}}}$$

17. For both LoD and LoQ, calculate the relative SD ($FI_{Sample\text{-}Blank}$), defined as the coefficient of variation (*CV%)* as:

$$\boldsymbol{CV}\% = \frac{\boldsymbol{SD(FI_{Sample-Blank})}}{\boldsymbol{FI_{Sample-Blank}}} \times \mathbf{100}$$

18. There is no specific value for a coefficient of variation that is considered to be a "good" value, because it depends on each specific experiment and your target *CV%* threshold. Nonetheless, it is common to categorize *CV%* as follows: excellent (≤10%), good (between 10% and 20%), acceptable (between 20% and 30%), and poor (>30%) [7,8]. Confirm that the *CV%* for LoQ is below 30%. If the *CV%* for LoQ exceeds 30%, it is recommended to repeat the experiment by increasing the cell concentration for LoQ (Table 2).

	**Statistical significance compared to Blank**	***CV%*≤ 30%**
Limit of Detection (LoD)	Yes	No
Limit of Quantification (LoQ)	Yes	Yes

Table S2. Criteria that LoD and LoQ must satisfy to be considered valid estimations.

Assessment of repeatability, reproducibility, and measurement uncertainty

Step 9:

1. Detach cells following the manufacturer's suggestions;
2. Resuspend cells in an appropriate volume of medium and count them;
3. Define at least 3 different cell concentration representing low, medium, and high confluency (e.g., 10%, 50%, and 90% confluency);
4. Seed the appropriate cell number in a multi-well plate (at least triplicate[2] for each condition is recommended). If possible, consider using a cell culture medium with low FBS concentration (e.g., 1-2% FBS) to avoid excessive cell growth;
5. Culture cells until they are firmly attached to the bottom of the plate using standard cell culture conditions (recommended time: 12-24 h).

Step 10:

6. Allow resazurin to reach RT. A 37°C water bath may be used to eventually thaw the reagent. Protect the resazurin from direct light;
7. Prepare the resazurin WS with a concentration between 40 and 50 µM in the complete cells culture medium and warm it at 37°C until use;
8. Remove the assay plate from the incubator and gently eliminate the medium from the wells;
9. Add an appropriate volume of resazurin WS in each well. The volume of resazurin WS depends on the multi-well plate used. For this experiment, a 96-well plate is recommended. Refer to Table 1 to select the correct resazurin WS volume;
10. Incubate cells using standard culture conditions (using previously optimized incubation time).
11. After incubation, gently remove the resazurin WS from the wells and transfer 100 µL in a 96-well plate for FI measurement;
12. Use a Fluorescence Microplate Reader to record the FI at the optimal λ_{Ex}-λ_{Em} combination previously selected;
13. Repeat steps 1-12 at least three times on three different days. Figure 6 summarizes the example of an experiment scheme;

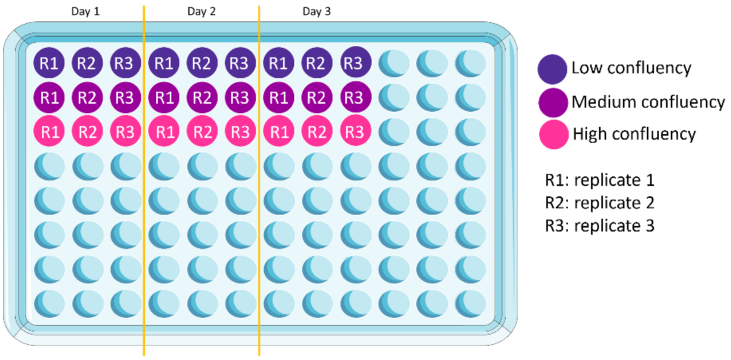

Figure S6. Example of an experimental scheme to assess repeatability, reproducibility, and MU

14. Calculate the FI_{mean} and SD of replicates for each confluency condition, for each day the experiment was performed;
15. Calculate repeatability for each confluency condition as [4]:

$$\boldsymbol{Repeatability} = \frac{(\boldsymbol{SD_{Day1}} + \boldsymbol{SD_{Day2}} + \cdots + \boldsymbol{SD_{Dayn}})}{\boldsymbol{n}}$$

n = times the experiment was repeated

16. For each confluency condition, calculate the relative repeatability, expressed as a percentage (%), as:

$$\boldsymbol{Relative\ Repeatability} = \frac{\boldsymbol{Repeatibility}}{\boldsymbol{FI}} \times \mathbf{100}$$

FI = FI_{mean} of all replicates for the confluency condition considered

17. Calculate reproducibility for each confluency condition as [4]:

$$\boldsymbol{Reproducibility} = \boldsymbol{SD_{All\ replicates\_All\ days}}$$

18. For each confluency condition, calculate the relative reproducibility, expressed as a percentage (%), as:

$$\boldsymbol{Relative\ Reproducibility} = \frac{\boldsymbol{Reproducibility}}{\boldsymbol{FI}} \times \mathbf{100}$$

FI = FI_{mean} of all replicates for the confluency condition considered

19. Evaluate the combined measurement uncertainty (*u*) from the experimental repeatability and reproducibility, following the equation [4]:

$$\boldsymbol{u} = \sqrt{\boldsymbol{repeatability}^2 + \boldsymbol{reproducibility}^2 + \boldsymbol{SD_{Vol}^2}}$$

SD_{Vol} = uncertainty related to pipette used to transfer metabolized resazurin WS in the 96-well for FI measurement [9–12]

20. Calculate the expanded uncertainty (*U*) according to the GUM [13], by multiplying *u* with a coverage factor *k* (e.g., *k*=2, 95% confidence level; *k*=3, 99% confidence level);
21. Calculate relative expanded uncertainty (U_{Rel}) as

$$\boldsymbol{U_{Rel}} = \frac{\boldsymbol{U}}{\boldsymbol{FI}} \times \mathbf{100}$$

FI = FI_{mean} of all replicates of the confluency condition considered

Evaluate the quality of the data considering that lower uncertainty corresponds to greater precision. Consider the purpose of your measurement, analyze data from the literature, if available, refer to industry standards and stakeholders to define acceptable limits or tolerances for a specific experiment.

Assays setup

Experiment setup

1. Seed an adequate number of cells in the multi-well plate chosen for the experiment. Optimal cell seeding density depends on cell type, experiment duration, and type (e.g., low cell confluency to test cell growth, medium/high confluency to test compound's cytotoxicity);
2. Carry out experimental conditions under which cellular viability is to be assessed. This involves, for example, adding growth factors, cytokines, or cytotoxic compounds to the culture media at various concentrations. It is advisable to include at least 5 dilutions of the substances of interest. In initial experiments, it is recommended to use a wide range of concentrations, subsequently narrowing it down to achieve more precise and accurate results;
3. The following control samples are recommended in each experiment [2]:
   i. No-Cell Control (Blank): resazurin WS only (wells without cells to serve as the negative control to determine FI background)[6];
   ii. Untreated Cells Control: wells with untreated cells to serve as a "starting point", for cell growth experiments, or as a "100% living cell point", for cytotoxicity tests. Add the same solvent used to deliver the test compounds;
   iii. Test Compound Control: wells without cells containing the solvent and compound to test for possible interference with resazurin chemistry;
   iv. Positive Control: wells containing cells treated with a known cell-proliferation-inducing compound for cell growth tests or a highly toxic substance in cytotoxicity tests;
   v. Empty wells (two or more) in the 96-well plate for FI detection: empty wells should display a minimal FI signal. This control sample is useful to verify that the plate reader is working correctly[7].
4. After culturing cells for the desired exposure period, allow resazurin to reach RT. A 37°C water bath may be used to eventually thaw the reagent. Protect the resazurin from direct light;
5. Prepare the resazurin WS with a concentration between 40 and 50 μM in the complete cells culture medium and warm it at 37°C until use;
6. Remove assay plates from the incubator and gently eliminate medium from the wells;
7. Add an appropriate volume of resazurin WS in each well (test and control wells). The volume of resazurin WS depends on the multi-well plate used. Refer to Table 1 to select the correct resazurin WS volume;
8. Incubate cells using standard culture conditions;
9. Reached the expected incubation time (previously optimized), gently remove the resazurin WS from the respective wells and transfer 100 μL in a 96-well plate for FI measurement;

[6] If cells are seeded onto or embedded in a scaffold, the No-Cell Control (Blank) will be the scaffold without cells with resazurin WS.
[7] This control sample is optional but recommended at least in the first experiments.

10. Use a Fluorescence Microplate Reader to record the FI at the optimal λ_{Ex}-λ_{Em} combination previously selected;
11. Calculate the FI_{mean} and SD of replicates for each test condition (test and controls wells);
12. Subtract the FI_{mean} of Blank from the FI_{mean} of all experimental wells ($FI_{Sample-Blank}$);
13. Calculate the SD ($FI_{Sample-Blank}$) by propagating the error using the formula::

$$SD(FI_{Sample-Blank}) = \sqrt{SD^2_{Sample} + SD^2_{Blank}}$$

14. Plot $FI_{Sample-Blank}$ (y-axis) versus compound concentration (x-axis).

Results quantification

Relative quantification

For proliferation assays, results are usually expressed as fold change (FC, a measure that describes how much a quantity changes between an original and a subsequent measurement [1]), normalizing results on "Untreated Cells Control" using the equation:

$$\boldsymbol{Cell\ growth_{FC}} = \frac{FI_{Compound}}{FI_{Untreat\ Cells\ Control}}$$

$FI_{Compound}$ = FI values of cells treated with different concentrations of compound

$FI_{Untreated\ Cells\ Control}$ = FI value of Untreated Cells Control

For cytotoxicity tests, results are usually expressed as percentage, normalizing on control samples by setting the Untreated Cells Control as "100% living cell point" and the Positive Control as "0% living cell point", applying the following formula ("min-max normalization" method, also called "feature scaling"):

$$z_s = \left(\frac{x_{s-\min(x)}}{\max(x) - \min(x)}\right) \times 100$$

z_s = normalized value of the sample in the dataset

x_s = value of the sample in the dataset

min(x) = the minimum value in the dataset (Positive Control)

max(x) = the maximum value in the dataset (Untreated Cells Control)

Absolute quantification

Calibration curve preparation:

1. In a range between 12 and 24 h before the conclusion of the experiment, detach cells following the manufacturer's suggestions (the same cell line(s) used for your experiment);
2. Resuspend cells in an appropriate volume of medium and count them;
3. Prepare at least 5 cell serial dilutions in a complete culture medium. The standard concentrations should at least cover the range of estimated concentrations for the unknown test samples and be evenly spaced throughout the range[8] [14]. If possible, consider using a cell culture medium with low FBS concentration (e.g., 1-2% FBS) to avoid excessive cell growth;
4. Seed the cell dilutions in a multi-well plate (at least triplicate[2] wells for each condition are recommended);
5. Culture cells until they are firmly attached to the bottom of the plate using standard cell culture conditions.

Cell concentration evaluation:

6. Simultaneously with the addition of resazurin WS to the test samples, treat the wells with the calibration curve equally;
7. Reached the expected incubation time (previously optimized), gently remove the resazurin WS from the wells and transfer 100 µL in a 96-well plate for FI measurement;
8. Use a Fluorescence Microplate Reader to record the FI at the optimal λ_{Ex}-λ_{Em} condition previously selected.
9. Calculate the FI_{mean} and SD of replicate for each test condition (calibration curve, unknown samples, and controls);
10. Subtract the FI_{mean} of Blank from the FI_{mean} of all experimental wells and calibration curve ($FI_{Sample-Blank}$);
11. Calculate the SD ($FI_{Sample-Blank}$) by propagating the error using the formula:

$$\boldsymbol{SD(FI_{Sample-Blank}) = \sqrt{SD^2_{Sample} + SD^2_{Blank}}}$$

12. For the calibration curve, plot $FI_{Sample-Blank}$ versus the number of cells seeded (e.g., cell n°/well or cell n°/cm^2);
13. Calculate the linear equation resulting from the calibration curve (Fig. 7). Check that R^2 is higher than 0.97. If R^2 is lower, repeat the experiment or exclude one or more points of the curve, avoiding having a calibration curve with less than 5 points;

[8] For a more convenient estimation of the calibration curve range, consult literature data, focusing on specific parameters relevant to the experiment type (e.g., cell duplication time under normal conditions, IC50, or LD50 of the tested compound).

14. Use the resulting linear equation to estimate the cell concentration of unknown samples by interpolating their $FI_{Sample\text{-}Blank}$ values (y values) (Fig. 7) as follows:

$$\boldsymbol{x = \frac{y - b}{m}}$$

x: cell concentration of the unknown sample

y: $FI_{Sample\text{-}Blank}$ of the unknown sample

b: y-intercept of the linear equation derived from the calibration curve

m: slope (S) of the linear equation derived from the calibration curve

15. If the cell concentration values obtained for unknown samples were outside the range of the calibration curve (Fig. 7, unknown sample "c"), it is recommended to repeat the experiment by varying the calibration curve dilution range.

N.B.: A calibration curve must be used in each independent experiment and must be incubated with resazurin WS under identical conditions as the unknown and control samples.

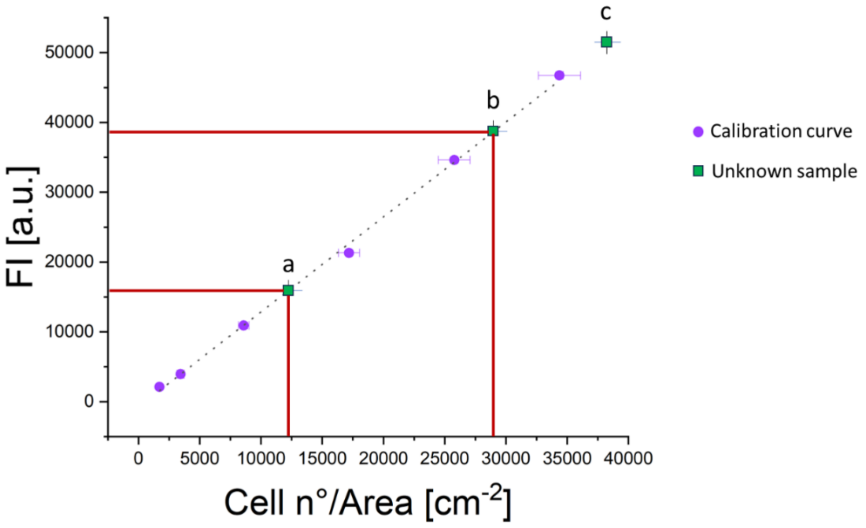

Figure S7. Example of absolute quantification result: $FI_{Sample\text{-}Blank}$ (y-axis) versus cell concentration (x-axis). Error bars on y-axis indicate SD, while those on x-axis indicate the cell counting uncertainty. Calibration curve points are indicated in purple, while unknown samples are represented in green. Unknown samples "a" and "b" can be interpolated with the linear equation derived from the calibration curve to estimate their cell concentration (x value, unknown) from FI measurement (y value, known); the cell concentration of unknown sample "c" should not be extrapolated because its FI value falls outside the range covered by the calibration curve.

Outlier management and significance assessment

Outliers

By carrying out all the steps described above, the possibility of obtaining outlier data is significantly reduced. However, the risk of addressing technical errors cannot be completely eliminated. Outliers can be considered and managed in several ways:

- Exclusion of extreme values from the analysis after a rational observation of the data distribution may be done based on the operator's experience;
- If possible, repeat the assay to validate the results. Outliers may be due to technical errors or variability that can be reduced with additional experiments;
- Consider the biological context and variability inherent in cell cultures. In some cases, biological outliers may reflect genuine variability rather than experimental error.

Outliers can be identified by:

- creating graphs or plots (e.g., scatter plots or box plots with the Tukey method) to visualize the distribution of data;
- utilizing statistical methods for outlier detection, such as the Z-score, Dixon's Q test, or Grubbs' test.

Statistically differences between data populations

If the difference between two data populations is within the U of your method, it suggests that the observed difference may not be statistically significant when compared to the uncertainty associated with your measurements. In other words, the difference could be due to random variability or measurement error rather than a true difference between the populations. In such cases, the observed difference may not be considered statistically significant.

Furthermore, biological significance should be considered: even if a difference is statistically significant, it's important to consider whether the observed difference is relevant from a biological perspective.

References

1. Borowski, E.J.; Borwein, J.M. Collins Dictionary of Mathematics. *Collins dictionary of Mathematics* 2012.
2. ASTM International F3504 − 21_Standard Practice for Quantifying Cell Proliferation in 3D Scaffolds by a Nondestructive Method 2021.
3. Lavogina, D.; Lust, H.; Tahk, M.-J.; Laasfeld, T.; Vellama, H.; Nasirova, N.; Vardja, M.; Eskla, K.-L.; Salumets, A.; Rinken, A.; et al. Revisiting the Resazurin-Based Sensing of Cellular Viability: Widening the Application Horizon. *Biosensors* **2022**, *12*, 196, doi:10.3390/bios12040196.
4. Divieto, C.; Sassi, M.P. A First Approach to Evaluate the Cell Dose in Highly Porous Scaffolds by Using a Nondestructive Metabolic Method. *Future Science OA* **2015**, *1*, fso.15.58, doi:10.4155/fso.15.58.
5. Chandran, S.; Singh, R.S.P. Comparison of Various International Guidelines for Analytical Method Validation. *Pharmazie* **2007**, *62*, 4–14.
6. Committee for Medicinal Products for Human Use ICH Guideline Q2(R2) on Validation of Analytical Procedures 2022.
7. Aronhime, S.; Calcagno, C.; Jajamovich, G.H.; Dyvorne, H.A.; Robson, P.; Dieterich, D.; Isabel Fiel, M.; Martel-Laferriere, V.; Chatterji, M.; Rusinek, H.; et al. DCE-MRI of the Liver: Effect of Linear and Nonlinear Conversions on Hepatic Perfusion Quantification and Reproducibility. *Magnetic Resonance Imaging* **2014**, *40*, 90–98, doi:10.1002/jmri.24341.
8. Beah, A.; Kamara, A.Y.; Jibrin, J.M.; Akinseye, F.M.; Tofa, A.I.; Adam, Adam.M. Simulating the Response of Drought–Tolerant Maize Varieties to Nitrogen Application in Contrasting Environments in the Nigeria Savannas Using the APSIM Model. *Agronomy* **2020**, *11*, 76, doi:10.3390/agronomy11010076.
9. Divieto, C.; Revel, L.; Sassi, G.; Sassi, M.P. Uncertainty Analysis of Cell Counting by Metabolic Assays. *J. Phys.: Conf. Ser.* **2013**, *459*, 012051, doi:10.1088/1742-6596/459/1/012051.
10. Revel, L.; Santiano, M. *RT 15/2022 Manual Micropipettes: Internal Procedure for Periodic Verification, Data Acquisition and Processing Software*; 2022;
11. Technical Committee ISO ISO 8655-2:2022: Piston-Operated Volumetric Apparatus-Part 2: Pipettes 2022.
12. Technical Committee ISO ISO 8655-6:2022: Piston-Operated Volumetric Apparatus - Par 6: Gravimetric Reference Measurement Procedure for the Determination of Volume 2022.
13. Working Group 1 of the Joint; Committee for Guides in Metrology (JCGM/WG 1) JCGM 100:2008. Evaluation of Measurement Data — Guide to the Expression of Uncertainty in Measurement 2008.
14. Barwick, V. Preparation of Calibration Curves - A Guide to Best Practice 2003.
